# Utility of the Zebrafish Model for Studying Neuronal and Behavioral Disturbances Induced by Embryonic Exposure to Alcohol, Nicotine, and Cannabis

**DOI:** 10.3390/cells12202505

**Published:** 2023-10-23

**Authors:** Adam D. Collier, Abdul R. Abdulai, Sarah F. Leibowitz

**Affiliations:** Laboratory of Behavioral Neurobiology, The Rockefeller University, New York, NY 10065, USA

**Keywords:** zebrafish, rodents, humans/clinical, embryonic drug exposure, alcohol, nicotine, cannabis, neuropeptides, brain/neuronal development, behavior

## Abstract

It is estimated that 5% of pregnant women consume drugs of abuse during pregnancy. Clinical research suggests that intake of drugs during pregnancy, such as alcohol, nicotine and cannabis, disturbs the development of neuronal systems in the offspring, in association with behavioral disturbances early in life and an increased risk of developing drug use disorders. After briefly summarizing evidence in rodents, this review focuses on the zebrafish model and its inherent advantages for studying the effects of embryonic exposure to drugs of abuse on behavioral and neuronal development, with an emphasis on neuropeptides known to promote drug-related behaviors. In addition to stimulating the expression and density of peptide neurons, as in rodents, zebrafish studies demonstrate that embryonic drug exposure has marked effects on the migration, morphology, projections, anatomical location, and peptide co-expression of these neurons. We also describe studies using advanced methodologies that can be applied *in vivo* in zebrafish: first, to demonstrate a causal relationship between the drug-induced neuronal and behavioral disturbances and second, to discover underlying molecular mechanisms that mediate these effects. The zebrafish model has great potential for providing important information regarding the development of novel and efficacious therapies for ameliorating the effects of early drug exposure.

## 1. Introduction

The consumption of drugs of abuse during pregnancy is a critical public health concern throughout the world and is known to increase the offspring’s risk of developing long-term changes in the brain that produce functional and behavioral disturbances. It is estimated that about 5% of pregnant women in the US use one or more addictive substance [1], with the most common being alcohol, nicotine and cannabis [2]. Elucidating the underlying neural mechanisms produced by prenatal drug exposure that drive behavioral disturbances in the offspring is important for discovering novel therapeutics which may help to mitigate the harmful effects of these substances that can appear early in development and persist throughout life. In this review, we first provide a brief overview of the human and rodent studies that investigate the effects of prenatal exposure to alcohol, nicotine, and cannabis, both on various behaviors related to drug intake and on the development of neurons that express specific peptides known to have a major role in controlling these behaviors. These peptides, including hypocretin/orexin (Hcrt), melanin-concentrating hormone (Mch), galanin (Gal), enkephalin (Enk), and dynorphin (Dyn), are heavily expressed in specific brain areas such as the hypothalamus, nucleus accumbens and amygdala that mediate drug consumption and other related behaviors that these peptides and areas control, including hyperactivity, anxiety, novelty seeking, exploration, impulsivity, and drug seeking, which are closely associated with the overconsumption of drugs.

**Figure 1:**
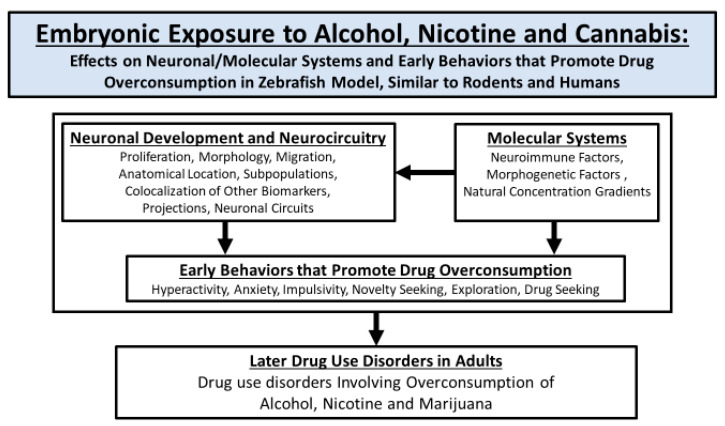
Schematic summarizing the main sections of this review, focusing on the use of zebrafish to study the effects of embryonic exposure to alcohol, nicotine and cannabis on neuronal and molecular systems and early behaviors that promote drug overconsumption. Advanced techniques available to study the direct relation of the brain to behavior in zebrafish after embryonic drug exposure include, for example, optogenetics, chemogenetics, targeted laser ablation, genetic manipulations, calcium imaging, and behavioral screenings. Examples of the use of some of these techniques in the context of embryonic alcohol exposure can be found in our recent studies [3,4,5,6,7].

Building on this evidence in humans and rodents, we then focus our attention on studies in zebrafish. After reviewing the many advantages of this animal model for investigating the effects of embryonic drug exposure, we describe in this species the variety of drug-induced disturbances in behaviors that appear early and in embryonic development of the peptide-expressing neurons and their circuitry, with these effects in many cases found to be similar to those observed in rats and even humans. We next summarize evidence for specific molecular mechanisms within the peptide-expressing neurons that mediate these prenatal drug-induced effects on their development and function in relation to behavior. These sections are then followed by a discussion of results, obtained using a variety of techniques, supporting the hypothesis that these disturbances in specific peptide neuronal systems are directly and possibly causally linked to the early changes in specific behaviors related to excess drug consumption. The final section summarizes the multiple approaches, which are more readily used in zebrafish than rodents or humans, that are needed in future studies of embryonic drug exposure to establish a close, functional relationship between the disturbances in peptide-expressing neurons, their molecular signaling, and the behavioral changes observed at different stages of life. Altogether, this review as summarized in Figure 1 demonstrates how the zebrafish is an excellent model for investigating in depth the brain mechanisms mediating the behavioral disturbances observed in multiple species after early exposure to drugs of abuse.

## 2. Human Studies of Prenatal Exposure to Drugs of Abuse

### 2.1. Advantages and Limitations of Human Studies

Studies of the effects of prenatal exposure to substances of abuse in human subjects have their own unique advantages as well as their limitations. Since the ultimate goal of this field of research is to discover efficacious methods for alleviating the harmful effects of prenatal exposure to drugs such as alcohol, nicotine and cannabis, developmental studies in human subjects clearly have the greatest potential for yielding the most relevant and useful information for treating and improving the human condition. However, clinical studies face a host of challenges and confounding factors. These include the complex environmental, hormonal and genetic variables that affect brain development, the widely variable timing and concentration of drug exposure along with polydrug use, and the often inaccurate self-reporting and diagnosis of affected children [8]. In addition, there are obvious practical issues with studying neuronal development in the brain of human embryos during drug exposure, and due to their correlative nature, brain imaging studies of the offspring after birth are limited in the amount of information they can generate regarding the functional relationship of the disturbances in neuronal systems to the behavioral changes in the offspring. Despite these issues, clinical studies are clearly informative and have made important discoveries regarding the variety of effects produced by prenatal drug exposure.

### 2.2. Behavioral Disturbances Observed in Human Studies

In addition to the multitude of negative health consequences produced by prenatal exposure to drugs of abuse including birth defects, small head circumference, and sudden infant death syndrome, studies of human offspring have demonstrated a wide range of behavioral disturbances, even in childhood that persist throughout life, including an increase in motivated behaviors, emotional and mood disorders, hyperactivity, impulsivity, risk-taking behavior and aggression, as well as cognitive issues [9,10,11,12]. Interestingly, when evident at a young age under natural conditions without prior drug exposure, these behavioral disturbances are shown to be predictive of a later increase in drug use [13,14,15,16,17,18,19]. Also, when stimulated by prenatal exposure to alcohol, nicotine or cannabis, they are similarly associated with an increased use of these drugs, often at an early age [20,21,22,23,24]. For example, prenatal exposure to alcohol causes babies to exhibit heightened reactivity towards alcohol odor, induces 18-year-olds to rank alcohol odors as particularly pleasant, and increases the abuse of alcohol in 22-year-olds [23,25,26]. Moreover, early age initiation of drug use is strongly associated with a later development of substance use disorders [27], with 15% of those who consume alcohol before the age of 14 compared to the 2.1% who wait until age 21 being more likely to develop an alcohol use disorder [28]. This evidence underscores the importance of identifying behavioral disturbances early in life that will allow therapeutic strategies to be enacted in a timely manner.

### 2.3. Neuronal Disturbances Observed in Human Studies

Although there are methodological restrictions limiting the investigation *in vivo* of neuronal changes in human subjects, clinical reports of fetal brain tissue and functional neuroimaging studies in children have provided evidence for alterations in different neuronal systems. For example, in the fetal forebrain, prenatal exposure to alcohol is shown to reduce mRNA expression of the Dyn kappa receptor that controls emotional and addictive behaviors [29,30,31] as well as alcohol consumption [32,33], and prenatal exposure to cannabis increases mRNA expression of the mu opioid receptor while reducing expression of Enk and also the dopamine D2 receptor that is positively correlated with the reported maternal cannabis use during pregnancy [31,34]. Also, pharmacological studies in humans show that opiates increase the number of Hcrt neurons in the brain [35], and antagonists of the Hcrt receptor have clinical efficacy in reducing withdrawal symptoms and improving sleep [36]. A modern *in vitro* brain organoid-on-a-chip technique using human pluripotent stem cells, which recapitulates key features of early brain development, has recently been employed to demonstrate that prenatal exposure to nicotine causes premature neuronal differentiation, induces abnormal migration, and alters cortical development [37]. Further, functional imaging has revealed changes in neuronal development after prenatal drug exposure that are associated with behavioral disturbances. For instance, MRI has demonstrated neural correlates of behavioral alterations in children, with changes in functional connectivity found to predict cognitive outcomes after prenatal alcohol exposure [38] and reduced volume of brain regions in adolescents with histories of heavy prenatal alcohol exposure found to be associated with reduced performance in a verbal memory test [39]. Together, these studies provide evidence for early, drug-induced disturbances in neuronal peptide systems and their connectivity in the human brain which may contribute to later disturbances in behaviors related to the overconsumption of addictive drugs.

## 3. Rodent Studies of Prenatal Exposure to Drugs of Abuse

### 3.1. Advantages and Limitations of Rodent Studies

Overcoming many of the limitations of clinical studies, rodents have been used to investigate how prenatal drug exposure affects the development of neurons as they relate to disturbances in behavior, with these animals having their own unique advantages and disadvantages as described in several reviews [12,40,41]. Briefly, compared to clinical studies, the major advantages of investigations in rats and mice include the experimenter’s ability to directly control the dose and timing of embryonic drug exposure as well as genetic and environmental variables and also to employ more invasive experimental manipulations that allow one to investigate more directly how changes in neuronal systems relate to the behavioral disturbances. Moreover, with a high degree of physiological, genetic, and neuronal conservation, rodent models are particularly helpful in identifying the underlying mechanisms and translationally relevant biomarkers of prenatal drug exposure.

As for their limitations, a notable one is that the timeline of neurogenesis in the rodent brain differs from that of humans, as does the timeline of their gestation, with that of rodents corresponding to the first and second trimester of humans and roughly the first 10 days of rodent life corresponding to the third trimester in humans [42,43,44]. Thus, modeling the third trimester effects of drug exposure on brain development requires substances to be directly administered to neonatal offspring using invasive methods and at a time when the pharmacokinetics differ markedly from those during in utero exposure [40]. Another challenge for the rodent studies is that they differ widely in the timing and dose of drug delivery and also in the many routes of drug administration, including subcutaneous, intravenous and intraperitoneal injections, oral gavage, osmotic minipumps, voluntary consumption, and smoke/vapor inhalation. This diverse methodology clearly challenges one’s ability to make comparisons across studies and to establish clear phenotypes of prenatal exposure to different drugs of abuse [12,40,45,46]. Despite these issues, studies in rats and mice have clearly generated important information for how prenatal drug exposure affects brain development and behavior, using methods that are difficult or impossible to employ in human studies.

### 3.2. Behavioral Disturbances Observed in Rodent Studies

Prenatal exposure to alcohol, nicotine, or cannabis in rodents has been shown to produce a wide range of behavioral changes in the offspring that may begin early in life and last long into adulthood, with parallel effects observed in human studies. There are several comprehensive reviews of the behavioral outcomes of prenatal exposure to these drugs of abuse [45,46,47,48], with some reports examining rodents at a young age [49,50,51,52,53,54]. In a recent study from our laboratory [4], we demonstrated in early postnatal rats that prenatal exposure to alcohol, at a relatively low concentration and for only 5 days, produces a number of drug-induced behaviors in the offspring, consistent with those described in clinical studies of children [26,55,56,57,58,59]. These early behaviors stimulated by alcohol exposure include an increase in locomotor activity, anxiety, exploration, and alcohol-seeking behavior, and they are followed by a later increase in voluntary alcohol consumption during adolescence, consistent with other reports showing these behaviors to persist into adulthood [46,60,61]. This increase in alcohol consumption at a young age, induced by alcohol administered during pregnancy at a low concentration that increases blood alcohol to levels < 150 mg/dL, suggests that the required preference for alcohol is a conditioned response that is established prenatally [62,63].

Notably, these drug-related behaviors are also produced by prenatal exposure to nicotine [45], and, consistent with clinical studies of nicotine dependence [64,65], they are associated in rodents with an increase in preference for nicotine odor during postnatal and adolescent ages [66] and also in the consumption and self-administration of nicotine in adolescent offspring [24,67]. Behavioral changes have also been observed in young rodents, as shown by altered locomotor activity in pre-weanling rats [68] and increased anxiety-like and impulsive behaviors in adolescent rats [69]. Prenatal exposure to cannabis in rodents has similar effects on drug-related behaviors [48,70,71], including a small increase in anxiety and impulsive risk-taking behavior during adolescence [72,73]. Further, long-lasting alterations in drug-seeking behavior have been shown after prenatal exposure to tetrahydrocannabinol (THC), the primary psychoactive molecule in cannabis that crosses the fetoplacental barrier [74], which include an increase in behavioral sensitivity to this drug in pre-adolescent rats [73] and also in heroin self-administration in adult rats [75]. These findings are consistent with clinical evidence, showing that exposure to cannabis early in life increases the use of other drugs [76,77] and that prenatal cannabis predicts an increased use of cannabis in adolescent and young adults [22,78]. Thus, despite the variation in behavioral outcomes due to a range of experimental factors, rodent studies clearly demonstrate that prenatal exposure to alcohol as well as nicotine and cannabis produce changes in drug-related behaviors that can be observed early in development and persist throughout life, in association with a later increase in consumption of these drugs of abuse.

### 3.3. Neuronal Disturbances Observed in Rodent Studies

Rodent models have been helpful in identifying a wide range of changes in neuronal systems after prenatal exposure to alcohol, nicotine and cannabis that are associated with alterations in behaviors. While most commonly studied at high doses that consistently impair neurogenesis and maturation of neuronal populations and synaptic plasticity and that produce neuronal apoptosis in different brain regions [47,79,80], prenatal exposure to alcohol at relatively low doses is found to have the opposite effects on neuronal development [24]. Specifically, studies in our laboratory [81] and others [82,83] demonstrate that alcohol stimulates cell proliferation and neurogenesis in specific brain areas, including the hypothalamus, nucleus accumbens, amygdala, thalamus, and hippocampus, while having no effect on gliogenesis and apoptosis. Prenatal alcohol exposure also increases the expression and proliferation of specific neurons containing the neuropeptides, Hcrt, Mch, Gal, Enk and Dyn [5,24,84,85,86], which are known to control a variety of behaviors such as arousal, motivation, reward, and numerous aspects of consummatory behavior that include the intake of and preference for alcohol and other drugs of abuse [24,87,88,89]. In addition to increasing the density of Hcrt and Mch neurons in the hypothalamus where they are normally located, our studies in early postnatal rats [84] demonstrate that prenatal exposure to alcohol at a relatively low concentration changes the morphology of these neurons [5], increasing both their size and number of their projections, similar to a report of altered morphology and projections of prefrontal pyramidal neurons after prenatal alcohol exposure in rats [90]. Thus, alcohol exposure at even low concentrations clearly alters the development of peptide neurons, likely resulting in functional changes that in turn drive changes in behavior.

Prenatal nicotine exposure is also found to affect the neuronal architecture and function of the hypothalamus. While at high doses peptide neurons may be reduced or unaffected [91], prenatal exposure to a low dose of nicotine, similar to alcohol, increases in pre-weanling offspring the density of Hcrt and Mch neurons in the hypothalamus and of Enk neurons in both the hypothalamus and amygdala, effects that persist until puberty [24,67,92,93]. Again, this increase in density of neurons is accompanied by a stimulation of neurogenesis with no change in gliogenesis or apoptosis, and also by an increased number of newly generated peptide-expressing neurons, effects followed by, and positively related to, a later increase in consumption of nicotine in addition to alcohol but not of lab chow or water. These findings are consistent with results of a study of Hcrt neurons which support a role for this peptide in multiple aspects of nicotine intake behavior, including the willingness to work and seek this rewarding substance and the emotional response associated with nicotine consumption [92]. While there are few studies in rodents of the effects of prenatal cannabis exposure on neuronal peptides, with one suggesting that prenatal cannabis exposure has no effect on cell proliferation in the dentate gyrus [94], there are reports suggesting that prenatal cannabis exposure alters the developmental route of several neuronal regions in the brain, with these effects correlated with functional consequences [48]. There is also evidence that prenatal exposure to THC stimulates mRNA expression of Enk in the amygdala of adults while reducing it in the nucleus accumbens early in development [75], and that adolescent exposure to THC stimulates the Dyn kappa receptor in the nucleus accumbens [95]. Together, these studies provide evidence for early, drug-induced disturbances in peptide neurons and their receptors in the rodent brain and suggest that these effects are associated with later disturbances in behaviors related to the overconsumption of addictive drugs.

## 4. Zebrafish Studies of Embryonic Exposure to Drugs of Abuse

### 4.1. Advantages and Limitations of Zebrafish Studies

Zebrafish have recently emerged as a promising animal model for studying the effects of embryonic exposure to drugs of abuse on the brain and behavior. They have a number of advantages as a model which make this species well suited to this field, as described in detail in several comprehensive reviews [40,96,97,98,99,100]. In addition to being a cost-effective model due to their small size and high fecundity, having well-conserved genomic and neuronal systems, and being suitable for high-throughput phenotypic drug screens [101,102], a particularly notable advantage of the zebrafish model over rodents is that their eggs are externally fertilized and develop outside the maternal organism. This allows one to examine the direct effects of drug exposure and remove the potentially confounding factors of maternal care that influence neuronal and behavioral functioning in rodents [103]. With this external development, drugs can be administered directly to and absorbed by a large number of developing embryos after immersing them in a bath solution, allowing precise control of the dose and duration of drug exposure. As in rodents, however, zebrafish have additional variables, including genetic strain and conditions of the water environment, which impact behavioral and neuronal responses and need to be controlled during embryonic drug exposure and considered when comparing results from different labs [104,105,106,107].

Studies of alcohol exposure in zebrafish show that the internal concentration within developing eggs after 2 h of exposure, which ranges from 4–10% of the alcohol concentration in the water as measured using an AM1 Alcohol Analyser (Analox Instruments, London, UK), correlates linearly with the alcohol bath concentration [108,109]. Also, while one cannot measure blood alcohol concentrations in zebrafish embryos, due to their lack of a developed circulatory system [110], alcohol levels in embryonic tissue can be precisely measured using headspace gas chromatography; they show the internal embryo concentrations at 24 h to be 25–35% of the bath concentration, independent of genetic background [111,112], producing a range generally equivalent to the blood alcohol concentration reported in mammalian studies. Thus, with exposure to the relatively low alcohol concentration (0.5% *v*/*v*) commonly used in the literature, which produces no change in gross morphology [113,114], blood alcohol concentration is found to be ~0.12 g/dL, which falls within the range (0.005–0.212 g/dL) of that observed in humans at birth who are prenatally exposed to alcohol [115]. While not as well studied as embryonic exposure to nicotine or cannabis, zebrafish can similarly be used to investigate the effects of these drugs on neuronal and behavioral development, since, along with a well-conserved circuitry, they contain at a young age neuronal targets of these drugs, including nicotinic acetylcholine [116] and endocannabinoid receptors [117], and are able to metabolize nicotine into cotinine in a similar way to humans [118].

As for studying the brain, zebrafish have another major advantage in being transparent as embryos and larvae and highly genetically tractable, allowing methods of transgenesis to be well established for labeling neuronal populations with fluorescent proteins [119,120,121]. Together, these features permit zebrafish to be tracked over time under a microscope using time-lapse live imaging and then examined for the effects of embryonic drug exposure on the *in vivo* development of neurons [122]. Of further importance is the fact that zebrafish start to display complex behaviors at a young age. This occurs as early as 4 days after fertilization, when they begin to swim freely, and automated behavioral tracking software can be used alongside neuronal imaging in the same animals to directly relate alterations in brain development to disturbances in a variety of drug-related behaviors, such as hyperactivity, anxiety, impulsivity, novelty and drug seeking, and consummatory behavior. With their rapid development and ability to be housed in a small space and large numbers, longitudinal studies can be performed with zebrafish with much greater ease and many more subjects than in rodents and humans [123]. Thus, zebrafish strike an ideal balance between practical simplicity and system complexity as an animal model [124]; these many characteristics of the zebrafish establish it as a useful species for elucidating how embryonic drug exposure affects the development of both neurons and behavior and also for determining whether these neuronal changes are in fact causally related to the behaviors.

### 4.2. Behavioral Disturbances Observed in Zebrafish Studies

Although not as well established as rodent behavioral models, the use of zebrafish to investigate the behavioral effects of embryonic drug exposure has grown increasingly popular, with behaviors related to drug intake largely paralleling those observed in rodent and human studies. This is particularly true for embryonic exposure to alcohol, which has been studied in greater depth than nicotine and cannabis and found to have strong effects on zebrafish behavior, as summarized in several comprehensive reviews [97,123,125,126,127,128]. As in rodent studies, embryonic drug exposure in zebrafish alters behaviors in a dose-related manner, with low doses often having stimulatory effects while high doses have suppressive effects. These biphasic responses have been reported for locomotor activity following embryonic exposure to alcohol [129], nicotine [130] and cannabinoids [131]. They have also been reported in other behavioral tests, with embryonic exposure to nicotine causing an increase in seeking behavior at a low concentration while producing nicotine avoidance at a high concentration [132]. Since zebrafish have another major advantage of allowing behaviors to be tested very early in development, further studies at just 5 days after fertilization have shown nicotine to increase anxiety-like behavior while reducing habituation to an acoustic startle stimulus [133,134] and have shown cannabinoids to have a similar effect on this startle response [135]. In a recent report from our laboratory [4], we have demonstrated in zebrafish of this young age additional behaviors after embryonic alcohol exposure at a low concentration which are related to drug intake and are also very similar to those produced by prenatal alcohol exposure in rodents [46,49,53,60,61] and humans [26,55,56,57,58,59] (Table 1). These behaviors include an increase in locomotor activity, anxiety, exploration, motor impulsivity, and novelty seeking, specific responses shown to be predictive of a later increase in voluntary alcohol consumption, as described in rats [136,137,138] and humans [11,139].

These early behavioral disturbances induced by embryonic drug exposure in zebrafish, as shown in rodents [46,60,61] and humans [23,26], are found to be long-lasting and to persist into older ages, with alterations, for example, shown in adult zebrafish in risk taking behavior after embryonic exposure to alcohol [123,165] or cannabinoids [114] and a startle response after embryonic exposure to nicotine [166]. One behavior that has not been as well studied in the zebrafish model is voluntary drug consumption that occurs later in development and is known to be increased in rodent and human offspring by prenatal exposure to drugs of abuse [20,23]. To examine this behavior, we developed in our lab a model of voluntary alcohol intake in adult as well as juvenile zebrafish that involves feeding them small cubes containing a mixture of alcohol with gelatin and brine shrimp, and by quantifying the number of bites they take of these alcohol–gelatin cubes we found their intake to be strongly positively correlated with blood alcohol concentration [140,146,167]. As in rats, both juvenile and adult zebrafish, after embryonic exposure to alcohol at a relatively low concentration (0.5% *v*/*v*), are found to exhibit an increase in the number of bites they take of the alcohol–gelatin but not of the control gelatin cubes. This indicates that embryonic alcohol exposure produces a long-lasting increase in the consumption of alcohol in zebrafish, similar to that shown in rodents and humans.

### 4.3. Neuronal Disturbances Observed in Zebrafish Studies

In addition to these changes in behavior, embryonic drug exposure in zebrafish produces disturbances in the development of neurons, which can be observed during early stages of life, are long-lasting, and are comparable to those reported in rodents, as well as humans (Table 2). Similar to the behavioral studies, the neuronal effects in zebrafish of embryonic exposure to alcohol have been investigated more than those of nicotine and cannabis, with the effects summarized in several reviews [98,99,125,127,128,168]. As in rodents, these drug-induced disturbances in neurons observed in zebrafish are found to vary with the dose, duration, and age of drug exposure, as well as the age of analysis and strain of zebrafish. For example, while moderate–high doses of embryonic alcohol exposure (1–3%) produce morphological abnormalities [169], along with a decrease in neuronal differentiation [170] and number of neurons and neural precursor cells [171], alcohol at a lower concentration (0.5%) with no morphological effects increases the proliferation and differentiation of neurons in the developing hypothalamus [172,173]. Also, whereas alcohol at 1% for 3–6 days increases the expression of dopamine and serotonin and their metabolites in the larval zebrafish brain [174], lower concentrations (0.25–0.5% for 2 h) administered at 24 h post fertilization reduces their levels in adults [175] but has no effect when administered later at 36–48 h post fertilization [126]. Further, embryonic alcohol exposure is found to reduce the monoamines and their metabolites in the AB zebrafish strain but to have no effect in the TU zebrafish strain [108]. There are zebrafish studies of the other drugs showing that embryonic exposure to nicotine reduces in multiple brain areas the expression of immediate early genes [133] and that embryonic cannabinoid exposure reduces in the forebrain the expression of glutamic acid decarboxylase, a marker of GABAergic neuronal differentiation [114]. While these neurotransmitter systems, and particularly the monoamines, are the most well studied in zebrafish with embryonic drug exposure [98,108,174], a number of other investigations focusing on the neuronal systems expressing the different peptides have yielded a variety of interesting findings, as described in detail below.

#### 4.3.1. Neuropeptide Neuronal Disturbances Observed in Zebrafish

As shown in rodents after embryonic drug exposure [67,84,188], neuropeptide systems in zebrafish are found to be involved in mediating a number of behaviors related to drug consumption [189,190,191]. These peptides, which are well conserved in zebrafish [192,193,194], as demonstrated for Hcrt [195], Mch [196], Gal [197,198], Enk [199] and Dyn [200], are shown to have a natural functional relationship with a number of behaviors, including locomotor activity [201,202], anxiety [203,204], the sleep/wake cycle [205,206], and consummatory behavior [146,207]. Investigations in our lab demonstrate that embryonic exposure to alcohol at relatively low concentrations has a range of effects on the development of these peptide-expressing neurons. In addition to showing changes in their expression, these studies using other measures of these neurons describe profound alterations in their number, proliferation, migration, anatomical location and colocalization of a second peptide, and show these effects to be long-lasting and even to vary across subpopulations [3,5,146,173]. We also find these effects to be sex-dependent, and considerably stronger in females than males [173], consistent with findings in rodents [49,84]. While there are a few neuropeptide studies in adult zebrafish showing the consumption of alcohol–gelatin to increase mRNA expression of Hcrt and Gal [167] and treatment with morphine to increase the expression of Dyn mRNA [208], most studies of the neuropeptides after embryonic drug exposure as well as under natural conditions have examined the embryo and larval zebrafish, likely due to their inherent advantages at these young ages.

Reports in our lab of embryonic exposure to alcohol at low doses demonstrate increased expression of Hcrt and Gal in the hypothalamus of adult zebrafish, indicating that alcohol, even at low concentrations, can have long-lasting effects on these peptide neurons [146]. In addition to showing that embryonic alcohol exposure increases the number of Hcrt neurons in embryonic and larval zebrafish, we have used time-lapse live imaging in Hcrt:EGFP transgenic zebrafish [205] and found that alcohol also alters the migration of these neurons and ultimately causes them to become ectopically expressed outside their normal location in the hypothalamus [3]. This is consistent with our recent findings in neonatal rats, showing that prenatal alcohol exposure alters the anatomical location of Hcrt and Mch neurons, causing them to be ectopically located outside the hypothalamus and further toward the anterior in the nucleus accumbens core and ventromedial caudate putamen, where they have not previously been observed in rats [5,209,210]. These results agree with other evidence from zebrafish, showing embryonic alcohol exposure to induce ectopic oxytocin and facial branchial motor neurons [211,212]. Interestingly, we also found that embryonic alcohol increases the lateral migration and number of Hcrt neurons and produces this effect in an asymmetric manner, mostly on the left side of the brain [205]. This is similar to other reports of asymmetries after alcohol exposure in the migration of neural crest cells in zebrafish [213], in addition to an increase on the left side both of hippocampal volume in mice [214] and hemisphere activity in humans, in response to drug cue-induced cravings [215,216]. Another study in zebrafish of embryonic drug exposure on peptide-expressing neurons shows that cocaine alters the expression of substance P in an age-dependent manner, with an increase throughout the brain at 24 h post fertilization, followed by a decrease at 48 h [217].

In our recent study [7], we demonstrate that subpopulations of Hcrt neurons are differentially sensitive to alcohol, with embryonic exposure increasing the number and proliferation of Hcrt neurons in the more anterior region of the hypothalamus but not the posterior region. Studies in rats similarly report anatomically specific effects of prenatal alcohol exposure, with increased proliferation of Enk neurons occurring in the core but not the shell of the nucleus accumbens [81,177]. Also, with Hcrt neurons known to be neurochemically and genetically heterogenous [218] and to co-express Dyn that also controls alcohol-related behaviors [190,219], we discovered that embryonic alcohol exposure alters their pattern of co-expression, causing the specific Hcrt neurons stimulated in the more-anterior but not posterior subpopulation to express relatively little Dyn [7]. It is notable that, although Hcrt and Dyn are co-transmitted from the same vesicles [220], they have inverse relationships, with Hcrt being excitatory and associated with reward and arousal and the stimulation of dopamine neurons [220,221] and Dyn being inhibitory and associated with depressive-like states and the inhibition of dopamine [222,223]. Thus, the reduced levels of Dyn observed in the anterior subpopulation that is more sensitive to alcohol are likely to be functionally altered, with the increase in excitatory signaling from Hcrt contributing to an increase in the different behaviors. These studies illustrate how the smaller brain of zebrafish compared to rodents allows for studies such as these to quantify and characterize with relative ease the entire neuronal population. They yield new information regarding the effects of embryonic drug exposure, not only on the expression and number of neurons but also on their migration, proliferation and anatomical location, the formation of neuronal subpopulations, and the colocalization of other biomarkers.

#### 4.3.2. Neuronal Morphology and Circuitry Disturbances Observed in Zebrafish

In addition to these effects of embryonic drug exposure on neurons, alterations in the development of neuronal morphology and neurocircuitry have also been described. In larval zebrafish and similarly in neonatal rats, our studies demonstrate that embryonic alcohol exposure alters the morphology of Hcrt neurons, causing normally located Hcrt neurons in alcohol-exposed animals to be larger in size and to have more processes emanating from the soma [5]. This is similar to other reports for rodents, demonstrating that prenatal alcohol exposure produces larger hypothalamic neurons and increases dendritic complexity [224,225].

To investigate in greater depth the effects of alcohol on the development of Hcrt circuitry, we next used live-imaging and Imaris 9.9.1 software to quantify in larval zebrafish how embryonic alcohol affects the development of the projections from Hcrt neurons [7]. In control fish, we observed natural differences in the morphology and direction of projections from the anterior and posterior subpopulations of hypothalamic Hcrt neurons. Whereas both have some short projections, the more-anterior Hcrt neurons have long ascending projections to anterior brain regions, with low numbers of branch points, terminal points and Sholl interactions, and the more-posterior Hcrt neurons have long descending projections to posterior brain regions, with high numbers of branch points, terminal points and Sholl intersections. In addition, our findings demonstrate that embryonic alcohol exposure affects primarily the long projections from these subpopulations, with the posterior subpopulation of neurons exhibiting an increased innervation of the locus coeruleus and raphe nucleus, rich in norepinephrine and serotonin, respectively, which are known to mediate locomotor activity and sleep/wake behaviors [194,226]. As for the ascending projections from the anterior subpopulation found to innervate the subpallium, our results show that embryonic alcohol causes these hypothalamic Hcrt neurons to become ectopically located further toward the anterior in the preoptic area and to exhibit increased innervation not only of the subpallium, a brain area known to contain Hcrt receptors and to be homologous with the mammalian basal ganglia [227], but also of the dorsal pallium, an area not normally innervated by Hcrt neurons at the larval stage [195] but shown to participate in drug responses [228]. These findings in zebrafish suggest that embryonic alcohol exposure causes premature maturation of the Hcrt neurocircuitry, similar to that reported for the hippocampal pyramidal circuitry in rats [229].

Studies of embryonic exposure to the other drugs of abuse have also described altered development of neuronal morphology and circuitry in zebrafish. For example, embryonic exposure to nicotine produces long-lasting alterations in the axonal pathfinding of secondary motor neurons, effects that persist until adulthood and are reversed by treatment with nicotinic receptor antagonists [179,230]. In addition, there is evidence that embryonic exposure to THC reduces the axon diameter of reticulospinal neurons in the hindbrain, while cannabinol exposure decreases the number of axonal branches of motor neurons [184,231,232]. Thus, with their less-complex neurocircuitry, relative to that in rodents and humans, these zebrafish brain studies illustrate how the effects of embryonic drug exposure on the development of neuronal circuits can be studied in depth and readily quantified *in vivo*.

### 4.4. Mechanisms Underlying Neuronal and Behavioral Disturbances in Zebrafish

To understand the molecular mechanisms mediating the neuronal and behavioral disturbances produced by embryonic exposure to drugs of abuse, zebrafish are a strong model, well suited for using genetic manipulations and pharmacological tools which provide information that may elucidate potential therapeutic methods for preventing these disturbances. One possible mediator of the effects of embryonic alcohol exposure on the development of peptide neurons and behavior is the neuroimmune system, with inflammatory chemokines such as Cxcl12 and its receptor Cxcr4 shown to be expressed within neurons [176] and involved in neuronal migration [233,234], proliferation [235,236,237] and the development of their projections [234,238,239,240]. Other potential underlying mechanisms are key signaling pathways, such as fibroblast growth factor (Fgf), which is known to play a major role in neuronal development and drug addiction [241], brain-derived neurotrophic factor (Bdnf), which has an important role in neuronal proliferation and differentiation [242], and sonic hedgehog (Shh) signaling, which is involved in embryogenesis and central nervous system formation [243].

#### 4.4.1. Brain Mechanisms Underlying Behavioral Disturbances in Zebrafish

Pharmacological and genetic studies in zebrafish have provided evidence supporting the role of chemokine systems in mediating the effects of embryonic drug exposure on behavior. For example, administration of an antagonist of the Cxcr4 receptor in the water prior to embryonic alcohol exposure prevents the alcohol-induced increase in locomotor activity and anxiety-like behaviors observed in larval zebrafish, supporting an important role for this chemokine system in mediating these behaviors [6]. This evidence from zebrafish is consistent with results in rats, showing that prenatal injection of Cxcl12, the chemokine ligand of Cxcr4, induces behaviors similar to prenatal alcohol exposure, including an increase in novelty-induced locomotor activity and anxiety [24], and that neuronal chemokines, including Cxcl12 and its Cxcr4 receptor, are markedly stimulated by prenatal alcohol exposure [176,177,244] and are involved in alcohol-related behaviors [24,245,246]. Also, it is notable that an antagonist of the Cxcr4 receptor is able to block the increase in locomotor activity and the conditioned place preference induced by adult exposure to cocaine [247].

There are other studies demonstrating the role of growth factors and important signaling pathways in the behavioral disturbances produced by embryonic drug exposure. The increase in risk-taking/anxiety-like behaviors induced by alcohol or cannabinoids in juvenile zebrafish is found to be blocked by the overexpression of *fgf8* or *shh* mRNA or the administration of a Shh agonist [164,165,248]. Also, the number of neurons positive for Bdnf is altered by embryonic alcohol exposure, an effect that is correlated with their deficits in social behavior [249], and embryonic exposure to fentanyl that increases Bdnf expression causes a decrease in social preference while increasing anxiety [250]. Growth and neurotrophic factors have similarly been shown in rodent studies to be affected by prenatal alcohol exposure and to mediate alcohol-induced deficits in morphology and behavior [84,251,252,253,254]; in clinical reports, after prenatal alcohol exposure, children are found to have elevated circulating levels of Fgf2 [178]. Together, these studies of the neuroimmune system and growth factors suggest potential therapeutic targets for mitigating the harmful effects on drug-related behaviors induced by prenatal drug exposure.

#### 4.4.2. Brain Mechanisms Underlying Neuronal Disturbances in Zebrafish

In addition to behavior, the molecular mechanisms underlying neuronal disturbances produced by embryonic drug exposure have also been investigated in zebrafish, using pharmacological and genetic tools. A report from our lab [172] shows that embryonic alcohol exposure at a relatively low concentration increases levels of *cxcl12a* and *cxcr4b* mRNA in whole embryos and increases the number of differentiated neurons and of *cxcl12a* and *cxcr4b* transcripts within these neurons in the embryonic hypothalamus; it also demonstrates that these effects of alcohol are blocked by pretreatment with a Cxcr4 antagonist, supporting an important role for this chemokine system in mediating the alcohol-induced increase in neuronal differentiation. This is further supported by studies in rodents, demonstrating that chemokines are expressed in Hcrt and Mch peptide neurons and that prenatal alcohol exposure similarly increases chemokine co-expression within these neurons [49,176]. Also, prenatal administration of the chemokines themselves mimics the alcohol-induced increase in peptide expression, while administration of chemokine neutralizing antibodies or antagonists prevents these effects of alcohol [255]. In another study of zebrafish [6], we tested the involvement of this chemokine system in the effects of embryonic alcohol exposure on the location of Hcrt neurons, and found that pretreatment with a Cxcr4 antagonist prevents the alcohol-induced formation of ectopic Hcrt neurons further toward the anterior in the preoptic area. By analyzing levels of the *cxcl12a* transcripts and internalized Cxcr4b receptors throughout the embryonic brain, we further discovered that they both form a natural posterior-to-anterior concentration gradient, with levels lowest in the posterior region of the hypothalamus, higher in the anterior region, and highest in the anterior telencephalon; embryonic alcohol exposure, which stimulates their density in all areas while maintaining these gradients, increases chemokine expression only in the more-anterior Hcrt neurons that are ectopically expressed, an effect blocked by pretreatment with a Cxcr4 antagonist. These results demonstrate that increased chemokine expression acting along natural gradients mediates the alcohol-induced anterior migration of the ectopic Hcrt neurons.

We most recently used genetic tools to test in zebrafish how Cxcl12 mediates the effects of embryonic alcohol exposure on the subpopulations of Hcrt neurons and their projections [7]. In this report, we demonstrate that the overexpression of *cxcl12a* mimics the stimulatory effects of alcohol on the more-anterior, ectopic Hcrt subpopulations and also on the long anterior projections from these ectopic Hcrt neurons, as well as the posterior projections from the more-posterior, normally located Hcrt neurons. In addition, knockdown of *cxcl12a* blocks each of these effects of alcohol, providing strong evidence for a direct role of this chemokine in mediating these effects on the development of the Hcrt neurons and their neurocircuitry. This idea is further supported by another zebrafish study, showing that Cxcr4 directs the posterior outgrowth of dorsal habenula neuron projections and that the disruption of Cxcr4 causes these projections to become ectopic [256]. Similarly, Cxcl12 and Cxcr4 have been found to correlate with the axonal pathway of gonadotropin-releasing hormone neurons, and knockdown of these chemokines causes these peptide neurons to become abnormally located and to exhibit ectopic axonal projections [234]. This provides additional evidence, suggesting that the neuroimmune system plays a key role in mediating neuronal development and that chemokines are important targets for mitigating the harmful effects of embryonic drug exposure.

Growth factors have also been shown to be an important mediator of the embryonic drug exposure effects on neuronal development. For example, the decrease in expression of glutamic acid decarboxylase, a marker of GABAergic neuronal differentiation induced in the zebrafish forebrain by embryonic exposure to alcohol or a cannabinoid agonist, is rescued by the overexpression of *fgf8* or *fgf19* mRNA [164,257]. Also, there is evidence that neuronal chemokine expression may be mediated by Fgf signaling, with disruption of *fgf8* found to alter the location of Cxcl12 and Cxcr4 in the zebrafish brain [256], suggesting that Fgf pathways acting through chemokine systems are potential targets for mitigating the disturbing effects of embryonic drug exposure on neuronal development. A recent study in rats from our lab supports this possibility [84], demonstrating that prenatal alcohol exposure that increases the density of hypothalamic Mch-expressing neurons also stimulates the expression of the *fgf2* and *fgfr1* receptors within these Mch neurons and that these effects of alcohol are mimicked by peripheral injection of Fgf2 itself in pregnant rats. Building on these findings showing Fgf signaling to be altered by embryonic drug exposure in both zebrafish and rodents and other evidence strongly implicating Fgf in addiction in both rodents and humans [258], further research using the zebrafish model should help to better evaluate the therapeutic potential of this neuronal system.

### 4.5. Direct Relation of Disturbances in Neurons to Changes in Zebrafish Behavior

Due primarily to the optical transparency and small size of zebrafish embryos and larvae, this species is ideally suited for determining the direct and possibly causal relation of the neuronal changes induced by drugs of abuse with the disturbances also produced in different behaviors. In this regard, several useful tools have emerged in recent years in the zebrafish field that can be applied *in vivo* during behavioral testing. These techniques include optogenetic and chemogenetic neuronal activation and silencing [226,259], calcium imaging [260,261], and neuronal laser ablation [7,262], and they also involve genetic manipulations with tools such as CRISPR [263,264] and morpholino oligonucleotides [265,266]. These techniques applied prior to behavioral testing can probe the contribution of specific neuronal populations, including those expressing neuropeptides and functionally related genes, to drug-induced disturbances in particular behaviors.

To date, these techniques involving targeted ablation, optogenetics and calcium imaging have primarily been used to link neuronal populations to natural behaviors in the absence of drug exposure, with only a few reports investigating the peptide-expressing neurons. These include a study of Hcrt neurons [267], showing that their genetic overexpression and optogenetic activation both increase locomotor activity and arousal and that these behavioral effects are blocked in zebrafish lacking dopamine beta-hydroxylase that converts dopamine to norepinephrine, indicating a major role for norepinephrine in mediating the effects. Further, by also using calcium imaging to test the neuronal activity of norepinephrine-expressing neurons in the locus coeruleus, this study demonstrates that optogenetic activation of Hcrt neurons stimulates their activity, confirming that this hindbrain region is an important target of the Hcrt neurocircuit. Another report for both zebrafish and mice [226], which used an inducible genetic ablation system to destroy the raphe that is rich serotonergic neurons, found that the ablated animals exhibit increased wakefulness and reduced sleep, while optogenetic activation of the raphe neurons has the opposite effect, a reduction in wakefulness and increase in sleep, supporting a role of the specific raphe serotonergic neurons in mediating these behaviors.

Although these techniques involving direct neuronal measurements and manipulations hold great promise in revealing a direct relationship of specific neuronal changes induced by embryonic drug exposure with particular drug-induced disturbances in behavior, there are only a few studies to date in zebrafish that have accomplished this. A recent report from our lab on larval zebrafish [5], which used laser ablation to eliminate specific Hcrt neurons after embryonic alcohol exposure followed by behavioral analysis, demonstrates that the alcohol-induced increase in locomotor activity is completely blocked by ablation of the specific, alcohol-induced ectopic Hcrt neurons in the preoptic area but not of the normally located hypothalamic Hcrt neurons, a finding that underscores the importance of the ectopic neuronal subpopulation in mediating the disturbances in locomotor behavior. In another recent report [4], we demonstrated that optogenetic activation of Hcrt neurons in zebrafish under control conditions produces behavioral changes similar to those induced by embryonic alcohol exposure, including an increase in locomotor activity, anxiety, exploration, motor impulsivity, novelty seeking, and alcohol-seeking in larval fish, and also in voluntary alcohol consumption in juvenile fish. These zebrafish studies are consistent with rodent studies using optogenetic and chemogenetic activation of Hcrt neurons, showing that these manipulations stimulate locomotion [268], anxiety [268,269], and impulsivity [270]. While this evidence provides initial support for a specific role of Hcrt neurons in mediating the behavioral disturbances induced by embryonic alcohol exposure, more studies using these advanced techniques are clearly needed in zebrafish to investigate the causal relationship between the different neuropeptide systems affected by drugs of abuse and the behavioral disturbances that promote increased consumption of addictive drugs.

In addition to Hcrt, the contribution of other neuronal systems to behavioral disturbances produced by embryonic drug exposure has also been investigated in zebrafish. For example, there is a study of the neuronal and behavioral effects of embryonic THC and cannabidiol exposure in zebrafish that used the recently developed calcium indicator CaMPARI [187], which undergoes a switch from green to red fluorescence after elevated intracellular calcium concentrations, thus providing an indication of neuronal activation in free-swimming zebrafish larvae [271]. This study [187] shows that both whole-brain neuronal activity and locomotor activity are decreased by embryonic exposure to THC and cannabidiol, and that this cannabidiol-mediated reduction in locomotor activity is partially blocked by antagonists of the cannabinoid receptor. Other reports using genetic tools have investigated the contributions of specific neuronal markers and signaling pathways to mediating the effects of embryonic drug exposure on neuronal development and behavior. For instance, a nicotine-induced increase in locomotor activity is blocked by morpholino knockdown of a nicotinic acetylcholine receptor that has no effect of its own on spontaneous motor activity [272]. While not yet investigated in the context of embryonic drug exposure, pharmacological agents that modulate epigenetic regulation of neuronal gene expression are also found to block nicotine-induced conditioned place preference and other drug-related behaviors in adult zebrafish [273], warranting further investigations into the role of epigenetics in the effects of embryonic drug exposure on behavior.

## 5. Conclusions

With the consumption of drugs of abuse during pregnancy occurring widely throughout the world and known to produce long-lasting changes in the brain that result in functional and behavioral disturbances, the use of animal models to elucidate the mechanisms underlying these developmental changes is essential for discovering new therapeutic compounds and interventions. With the zebrafish being an especially advantageous model for this purpose and with great promise of yielding novel findings, more research in this species is clearly needed to directly relate drug-induced neuronal disturbances to alterations in behavior. This is especially true in relation to the peptide-expressing neurons, which to date have been relatively understudied in zebrafish, despite strong evidence in rodents showing them to be very sensitive to the effects of embryonic drug exposure and to mediate early drug-related behaviors that promote later overconsumption of the drug. Further, with a range of drug concentrations and developmental exposure times permitted in zebrafish, more studies measuring drug absorption and metabolism rates after embryonic exposure are needed to establish well-validated exposure paradigms that accurately model drug uptake in humans during gestation. Together with the advanced techniques that permit one to directly observe and manipulate neuronal development in the embryo, one can use high-throughput behavioral drug screening methods that target specific neuronal systems and thus hold great promise in identifying medications that may decrease behavioral deficits produced by embryonic exposure to drugs of abuse [130]. Thus, with recent evidence showing embryonic drug exposure in zebrafish to produce long-lasting changes in neuronal development and behavior similar to those demonstrated in rodents and in some cases in humans, the zebrafish model, with its notable advantages over rodents, has only begun to be harnessed to better understand not only how these neuronal and behavioral changes occur and are related to each other but also how the intracellular molecular mechanisms function to mediate these changes.

## Figures and Tables

**Table 1:** Effects of prenatal or embryonic exposure to alcohol, nicotine or cannabis in humans, rodents and zebrafish on different behaviors shown to be related to drug intake.

Drug Intake-Related Behaviors				
Drug	Behaviors	Human	Rodent	Zebrafish
Alcohol	Hyperactivity/Locomotor Activity	↑ [14]	↑ [51] ↓ [49]	↑ [140] ↓ [141]
	Anxiety/Startle Response	↑ [142]	↑ [50] ↑ [61]	↑ [140] ↓ [143]
	Impulsivity	↑ [11]	↑ [144]	
	Novelty Seeking/Risk Taking/Exploration	-	↑ [145] ↑ [53]	↑ [146]
	Drug Seeking/Intake	↑ [147] ↑ [139]	↑ [49]	↑ [140]
Nicotine	Hyperactivity/Locomotor Activity	↑ [148]	↑ [149]	↑ [118]
	Anxiety/Startle Response	↑ [150] ↑ [151]	↑ [152] ↑ [153] ↓ [72]	-
	Impulsivity	-	↑ [154]	-
	Novelty Seeking/Risk Taking/Exploration	-	↑ [155] ↓ [156]	-
	Drug Seeking/Intake	-	-	-
Cannabis	Hyperactivity/Locomotor Activity	↑ [157]	↑ [158]	↑ [131]
	Anxiety/Startle Response	↑ [159]	↑ [160]	-
	Impulsivity	↑ [161] ↑ [162]	-	-
	Novelty Seeking/Risk Taking/Exploration	-	↑ [163]	↑ [164]
	Drug Seeking/Intake	↑ [78]	↑ [75]	-

Abbreviations: ↑: increase; ↓: decrease; -: no results.

**Table 2:** Effects of prenatal or embryonic exposure to alcohol, nicotine or cannabis in human, rodents and zebrafish on brain neuronal populations expressing neuropeptides and other biomarkers.

Neuronal Effects				
Drug	Neuronal Population	Human	Rodent	Zebrafish
Alcohol	Hcrt	-	↑ Expression [176] ↑ Density [81] ↑ Proliferation [81] ↑ Ectopic Location [5] ↑ Projections [5] ↑ Morphology [5]	↑ Number [3,173] ↑ Proliferation [7] ↑ Asymmetry [3,173] ↑ Migration [3] ↑ Ectopic Location [3,5] ↑ Subpopulations [6,7] ↑ Projections [5] ↑ Morphology [5]
	Mch	-	↑ Expression [176] ↑ Density [177] ↑ Proliferation [177] ↑ Ectopic Location [5] ↑ Subpopulations [84] ↑ Projections [5] ↑ Morphology [5]	-
	Dyn	0 effect [31]	↑ Expression [86]	↑ Colocalization w/ Hcrt [7]
	KOR	↓ Expression [31]	-	-
	Enk	-	↑ Density [81] ↑ Proliferation [81]	-
	Gal	-	↑ Density [81] ↑ Proliferation [81]	↑ Density [146]
	Cxcl12	-	↑ Expression [176]	↑ Expression [172] ↑ Density [6,172] ↑ Colocalization w/ Hcrt [6]
	Cxcr4	-	↑ Expression [176] ↑ Density [176] ↑ Colocalization w/ Hcrt [176] ↑ Colocalization w/ Hcrt [176] ↑ Colocalization w/ Radial Glia [176]	↑ Expression [172] ↑ Density [6,172] ↑ Colocalization w/ Hcrt [6]
	Fgf	↑ Levels [178]	↑ Expression ↑ Colocalization w/ Mch [176]	-
	Gad 1	-	-	↓ Expression [114]
Nicotine	Hcrt	-	↑ Expression [67] ↑ Density [67] ↑ Proliferation [67]	-
	Mch	-	↑ Expression [67] ↑ Density [67] ↑ Proliferation [67]	-
	Enk	-	↑ Expression [67] ↑ Density [67] ↑ Proliferation [67]	-
	Motor Neurons	-	-	↑ Morphology [179] ↑ Axon Pathfinding Errors [179]
	Immediate Early Genes	-	↓ Expression [180]	↓ Expression [133]
	Gad 67	-	↑ Expression [181]	-
	Human Induced Pluripotent Stem Cells	↑ Differentiation [37]	-	-
Cannabis	Dyn	0 Effect [31]	-	-
	Enk	↓ Expression [31]	↑ ↓ Expression [75]	-
	KOR	↓ Expression [31]	↑ Expression [95]	-
	Mu Receptor	↑ Expression [31]	-	-
	CBR1	0 Effect [182]	↓ Expression [183]	
	Motor Neurons	-	↓Development [183]	↓Branching [184]
	Whole Brain	↓ Brain Size [185] ↓ Brain Volume [186]	-	↓ Neural Activity [187]
	Mauthner Cells	-	-	↑ Morphology [187] ↓ Axon Diameter [187]
	D2 Receptor	↓ Expression [182]	-	-
	Gad 1	-	-	↓ Expression [114]

Abbreviations: ↑: increase; ↓: decrease; No results; -; w/: with; Hcrt: hypocretin/orexin; Mch: melanin-concentrating hormone; Dyn: dynorphin; Enk: Enkephalin; Gal: galanin; Cxcl12: c-x-c motif chemokine ligand 12; Cxcr4: c-x-c chemokine receptor type 4; Fgf: fibroblast growth factor; Gad1: glutamate decarboxylase 1; Gad67: glutamate decarboxylase 67; KOR: k-opioid receptor; CBR1: cannabinoid receptor 1; D2: dopamine 2 receptor.

## Data Availability

Not applicable.
